# *Agave REVEILLE1* regulates the onset and release of seasonal dormancy in *Populus*

**DOI:** 10.1093/plphys/kiac588

**Published:** 2022-12-22

**Authors:** Degao Liu, Dan Tang, Meng Xie, Jin Zhang, Longmei Zhai, Jiangping Mao, Chao Luo, Anna Lipzen, Yu Zhang, Emily Savage, Guoliang Yuan, Hao-Bo Guo, Dimiru Tadesse, Rongbin Hu, Sara Jawdy, Hua Cheng, Linling Li, Huseyin Yer, Miranda M Clark, Huayu Sun, Jiyuan Shi, Roshani Budhathoki, Rahul Kumar, Troy Kamuda, Yanjun Li, Christa Pennacchio, Kerrie Barry, Jeremy Schmutz, Rajiv Berry, Wellington Muchero, Jin-Gui Chen, Yi Li, Gerald A Tuskan, Xiaohan Yang

**Affiliations:** Biosciences Division, Oak Ridge National Laboratory, Oak Ridge, Tennessee 37831, USA; U.S. DOE-Center for Bioenergy Innovation (CBI), Oak Ridge National Laboratory, Oak Ridge, Tennessee 37831, USA; Department of Genetics, Cell Biology and Development, University of Minnesota, Saint Paul, Minnesota 55108, USA; Department of Plant Science and Landscape Architecture, University of Connecticut, Storrs, Connecticut 06269, USA; National Center for Citrus Improvement, College of Horticulture, Hunan Agricultural University, Changsha 410128, Hunan, China; Biosciences Division, Oak Ridge National Laboratory, Oak Ridge, Tennessee 37831, USA; U.S. DOE-Center for Bioenergy Innovation (CBI), Oak Ridge National Laboratory, Oak Ridge, Tennessee 37831, USA; Biology Department, Brookhaven National Laboratory, Upton, New York 11973, USA; Biosciences Division, Oak Ridge National Laboratory, Oak Ridge, Tennessee 37831, USA; U.S. DOE-Center for Bioenergy Innovation (CBI), Oak Ridge National Laboratory, Oak Ridge, Tennessee 37831, USA; Department of Plant Science and Landscape Architecture, University of Connecticut, Storrs, Connecticut 06269, USA; Department of Plant Science and Landscape Architecture, University of Connecticut, Storrs, Connecticut 06269, USA; Department of Plant Science and Landscape Architecture, University of Connecticut, Storrs, Connecticut 06269, USA; U.S. Department of Energy Joint Genome Institute, Lawrence Berkeley National Laboratory, Berkeley, California 94720, USA; U.S. Department of Energy Joint Genome Institute, Lawrence Berkeley National Laboratory, Berkeley, California 94720, USA; U.S. Department of Energy Joint Genome Institute, Lawrence Berkeley National Laboratory, Berkeley, California 94720, USA; Biosciences Division, Oak Ridge National Laboratory, Oak Ridge, Tennessee 37831, USA; U.S. DOE-Center for Bioenergy Innovation (CBI), Oak Ridge National Laboratory, Oak Ridge, Tennessee 37831, USA; Materials and Manufacturing Directorate, Air Force Research Laboratory, Wright-Patterson Air Force Base, Dayton, Ohio 45433, USA; UES Inc., Dayton, Ohio 45432, USA; Biology Department, Brookhaven National Laboratory, Upton, New York 11973, USA; Biosciences Division, Oak Ridge National Laboratory, Oak Ridge, Tennessee 37831, USA; Biosciences Division, Oak Ridge National Laboratory, Oak Ridge, Tennessee 37831, USA; U.S. DOE-Center for Bioenergy Innovation (CBI), Oak Ridge National Laboratory, Oak Ridge, Tennessee 37831, USA; Biosciences Division, Oak Ridge National Laboratory, Oak Ridge, Tennessee 37831, USA; Biosciences Division, Oak Ridge National Laboratory, Oak Ridge, Tennessee 37831, USA; Department of Plant Science and Landscape Architecture, University of Connecticut, Storrs, Connecticut 06269, USA; Biosciences Division, Oak Ridge National Laboratory, Oak Ridge, Tennessee 37831, USA; U.S. DOE-Center for Bioenergy Innovation (CBI), Oak Ridge National Laboratory, Oak Ridge, Tennessee 37831, USA; Department of Plant Science and Landscape Architecture, University of Connecticut, Storrs, Connecticut 06269, USA; Department of Plant Science and Landscape Architecture, University of Connecticut, Storrs, Connecticut 06269, USA; Department of Plant Science and Landscape Architecture, University of Connecticut, Storrs, Connecticut 06269, USA; Department of Plant Science and Landscape Architecture, University of Connecticut, Storrs, Connecticut 06269, USA; Department of Plant Science and Landscape Architecture, University of Connecticut, Storrs, Connecticut 06269, USA; Department of Plant Science and Landscape Architecture, University of Connecticut, Storrs, Connecticut 06269, USA; U.S. Department of Energy Joint Genome Institute, Lawrence Berkeley National Laboratory, Berkeley, California 94720, USA; U.S. Department of Energy Joint Genome Institute, Lawrence Berkeley National Laboratory, Berkeley, California 94720, USA; U.S. Department of Energy Joint Genome Institute, Lawrence Berkeley National Laboratory, Berkeley, California 94720, USA; HudsonAlpha Institute for Biotechnology, 601 Genome Way, Huntsville, Alabama 35801, USA; Materials and Manufacturing Directorate, Air Force Research Laboratory, Wright-Patterson Air Force Base, Dayton, Ohio 45433, USA; Biosciences Division, Oak Ridge National Laboratory, Oak Ridge, Tennessee 37831, USA; U.S. DOE-Center for Bioenergy Innovation (CBI), Oak Ridge National Laboratory, Oak Ridge, Tennessee 37831, USA; Biosciences Division, Oak Ridge National Laboratory, Oak Ridge, Tennessee 37831, USA; U.S. DOE-Center for Bioenergy Innovation (CBI), Oak Ridge National Laboratory, Oak Ridge, Tennessee 37831, USA; Department of Plant Science and Landscape Architecture, University of Connecticut, Storrs, Connecticut 06269, USA; Biosciences Division, Oak Ridge National Laboratory, Oak Ridge, Tennessee 37831, USA; U.S. DOE-Center for Bioenergy Innovation (CBI), Oak Ridge National Laboratory, Oak Ridge, Tennessee 37831, USA; Biosciences Division, Oak Ridge National Laboratory, Oak Ridge, Tennessee 37831, USA; U.S. DOE-Center for Bioenergy Innovation (CBI), Oak Ridge National Laboratory, Oak Ridge, Tennessee 37831, USA

## Abstract

Deciduous woody plants like poplar (*Populus* spp.) have seasonal bud dormancy. It has been challenging to simultaneously delay the onset of bud dormancy in the fall and advance bud break in the spring, as bud dormancy, and bud break were thought to be controlled by different genetic factors. Here, we demonstrate that heterologous expression of the *REVEILLE1* gene (named *AaRVE1*) from *Agave* (*Agave americana*) not only delays the onset of bud dormancy but also accelerates bud break in poplar in field trials. *AaRVE1* heterologous expression increases poplar biomass yield by 166% in the greenhouse. Furthermore, we reveal that heterologous expression of *AaRVE1* increases cytokinin contents, represses multiple dormancy-related genes, and up-regulates bud break-related genes, and that *AaRVE1* functions as a transcriptional repressor and regulates the activity of the *DORMANCY-ASSOCIATED PROTEIN 1* (*DRM1*) promoter. Our findings demonstrate that *AaRVE1* appears to function as a regulator of bud dormancy and bud break, which has important implications for extending the growing season of deciduous trees in frost-free temperate and subtropical regions to increase crop yield.

## Teaser

*Agave REVEILLE1* regulates bud dormancy and bud break in *Populus* by repressing multiple dormancy-related genes and up-regulating bud break-related genes.

## Introduction

Woody plants provide humanity with many benefits such as wood, fiber, biofuels, and food (fruit/nuts) (Tuskan et al., 2006; Ragauskas et al., 2014; Li et al., 2019). Many of the woody plants are deciduous, modulating their annual growth and dormancy cycles (i.e. terminal and lateral buds become dormant in late fall followed by the release of dormancy in the spring) in response to seasonal changes in daylength and temperature (Ding and Nilsson, 2016; Miskolczi et al., 2019; Ramos-Sánchez et al., 2019; Maurya et al., 2020). Several bud dormancy-related genes (Böhlenius et al., 2006; Tylewicz et al., 2018; Busov, 2019; Singh et al., 2019) or bud break-related genes (Mohamed et al., 2010; Yordanov et al., 2014; Singh et al., 2018; Azeez et al., 2021) have been identified in trees. However, to date, single genetic element or master regulator that delays the onset of bud dormancy in the fall and advances bud break in the spring has not been identified. It is currently thought that bud dormancy and bud break are regulated by different molecular mechanisms (Ding and Nilsson, 2016).

Multiple *REVEILLE* (*RVE*) genes, which encode MYB-like transcription factors, have been up-regulated around the time of dormancy release in sweet cherry (*Prunus avium* L.) (Vimont et al., 2019) and during the chilling treatment of apples (*Malus domestica*) which is required for bud break (Porto et al., 2015). In addition, an *RVE* in Arabidopsis (*Arabidopsis thaliana*), called *AtRVE1*, was shown to be involved in promoting primary seed dormancy (Jiang et al., 2016). In our recent research, we identified an *RVE* gene in *Agave* (*Agave americana*), named *AaRVE1*, through transcriptome analysis, and predicted that *AaRVE1* acts as a circadian clock output that is entrained by the input signal (photoperiod) from environment (Abraham et al., 2016; Yin et al., 2018). Here, we report on the impact of ectopic expression of *AaRVE1* on the bud dormancy and bud break in poplar “717-1B4” (*Populus tremula* × *alba* clone INRA 717-1B4), and we demonstrated that *AaRVE1* appears to function as a regulator of bud dormancy and bud break.

## Results

**Figure 1:**
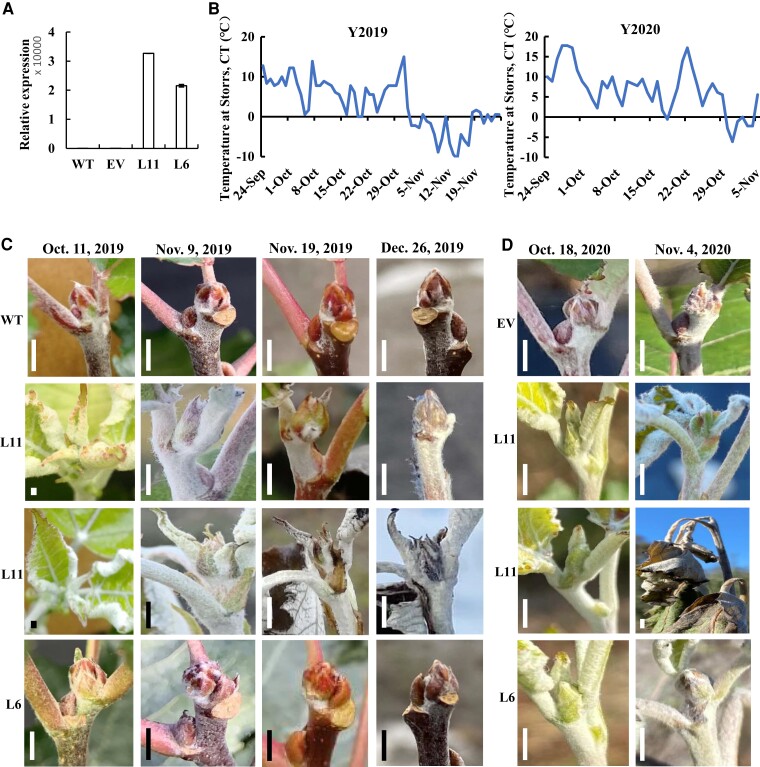
Heterologous expression of *AaRVE1* delayed seasonal dormancy in poplar in the field. A, Reverse transcription quantitative PCR (RT-qPCR) analysis to detect the expression of *AaRVE1* in transgenic plants expressing *AaRVE1* (L11, L6), empty vector (EV), and wild-types (WT). *Actin* gene was used as an internal control. Data are presented as mean ± Sd (*n* = 2). B, Lowest daily temperature in the field of Storrs, CT (41.8048° N, 72.2930° W). C and D, Apical phenotypes of the transgenic plants and WT in the field. Bar = 3 mm. In the year of 2019, all the L6 plants (9/9) showed slightly delayed dormancy compared with controls, while all the L11 plants (6/6) continued growing until early November when the temperature dropped below 0°C. The terminal bud of L11 gradually turned into dormancy-like phenotype in the middle of November. In the year of 2020, all the EV plants (6/6) turned into dormancy and stopped growing around October 18, while 42.9% (3/7) of L6 plants and 66.7% (4/6) of L11 plants continued growing until the snowfall and cold temperature (−6.1°C) on October 31.

The *AaRVE1* coding sequence was cloned, downstream of the cauliflower mosaic virus 35S (CaMV35S) promoter, into the binary vector pBI121 to yield p35S:*AaRVE1* (Supplemental Figure 1). The p35S:*AaRVE1* construct and empty vector (EV) (pBI121) were engineered into poplar “717-1B4” using *Agrobacterium*-mediated transformation. Two independent transgenic lines (L6 and L11) exhibiting different expression levels of *AaRVE1* (Figure 1A) were selected as representative lines for subsequent analyses, together with EV, and wild-type (WT) controls.

### Heterologous expression of the single *AaRVE1* gene delays the onset of bud dormancy during a 2-year field trials

A 2-year field trials was carried out in the University of Connecticut Depot Campus Field, Storrs, CT (41.8048° N, 72.2930° W). Transgenic, EV, and WT plants were planted in the field on August 15, 2019 (Supplemental Figure 2) and July 28, 2020. When the temperature dropped to 10°C and day length decreased to less than 12 h during late September and early October 2019 (Figure 1B; Supplemental Figure 2), all the WT (8/8) and EV (3/3) plants gradually entered dormancy and stopped growing (Figure 1C). In the year of 2019, the transgenic line L6 (9/9) showed a slight delay in dormancy compared with the controls while all the plants of transgenic line L11 (6/6), in which the expression level of *AaRVE1* was higher than that of line L6, continued growing until early November when the temperature dropped below 0°C (Figure 1C). The terminal buds of L11 gradually produced a dormancy-like phenotype in the middle of November. The apical buds and stem, in lengths of 2.0–8.5 cm (Counting from the shoot tip), of all the L11 plants were killed by subfreezing temperatures in the winter of 2019. In 2020, all the EV plants (6/6) went dormant and stopped growing around October 18, while 42.9% (3/7) of L6 plants and 66.7% (4/6) of L11 plants continued growing until the arrival of snowfall and cold temperature (−6.1°C) on October 31 (Figure 1, B and D).

The height of the WT and EV control plants on November 5, 2019 was 1.2-fold greater than their initial height, while the L11 plants were 2.7-fold taller relative to their initial height (Supplemental Figure 3). The height of L11 increased 4 cm from October 11th to November 5th while no growth was observed in WT and EV plants. The L11 also exhibited significantly greater stem diameter and internode length than WT (Supplemental Figure 3). These results establish that heterologous expression of *AaRVE1* substantially delayed bud dormancy and accelerated the growth in poplar in the fall. In the year of 2020, the height of L11 and L6 plants increased 7.6 and 3.5 cm on average from October 3rd to October 18th, respectively, while the height of EV increased 0.8 cm only (Supplemental Figure 4). No significant difference (Statistical significance was determined using two-tailed Student's *t*-test. *P* < 0.05 was considered statistically significant.) in the ratio of growth increase was observed between the transgenic *AaRVE1* plants and EV plants on October 18th. The lowest daily temperature during the end of September 2020 was much higher than that in the end of 2019. For example, the lowest daily temperature on September 27th of 2020 was 17.8°C while the lowest daily temperature on September 27th of 2019 was 7.8°C (Figure 1B). EV plants in the fall of 2020 delayed the onset of bud dormancy compared to the year 2019, which could reduce the difference in ratio of growth increase between the transgenic *AaRVE1* plants and EV.

### Heterologous expression of the single *AaRVE1* gene accelerates the bud break

**Figure 2:**
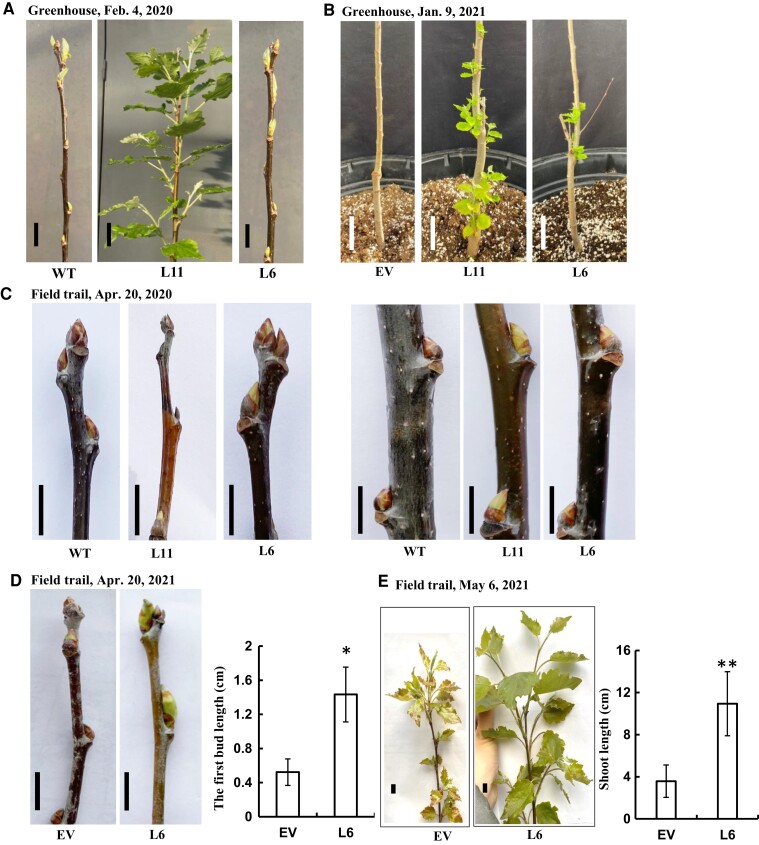
Heterologous expression of *AaRVE1* accelerated bud break in both greenhouse and field. A, Accelerated bud break of the transgenic plants expressing *AaRVE1* (L11, L6) in the greenhouse at the University of Connecticut. Plants were moved into the greenhouse on December 26, 2019. Photos were taken on February 4, 2020 in the greenhouse. Bars = 2 cm. WT: wild-type. B, Accelerated bud break of the transgenic plants expressing *AaRVE1* in the greenhouse at the University of Connecticut. Plants were moved into the greenhouse on December 2, 2020. The temperature range in greenhouse was 21°C–23°C under a 16 h light/8 h dark photoperiod. Photos were taken on January 9, 2021 in the greenhouse. Bars = 5 cm. EV: transgenic plants expressing the empty vector. C, Apical buds and middle-upper part of poplar grown in the field. The apical buds of L11 were frozen to death in the winter of 2019. Photos were taken on April 20, 2020. Bars = 1 cm. D, The first buds (counting from the top) of poplar (L6 and EV) grown in the field in the spring of 2021. The upper buds and stems of L11 were frozen to death in the winter of 2020. Photos were taken on April 20, 2021. Bars = 1 cm. EV: transgenic plants expressing the EV. L6: transgenic plants expressing *AaRVE1*. EV, *n* = 4; L6, *n* = 3. E, The average length of the 1st, 2nd, and 3rd shoots (counting from the top) formed on the plants grown in the field in the spring of 2021. Photos were taken on May 6, 2021. Bars = 1 cm. EV, *n* = 4; L6, *n* = 3. All the data in this figure are presented as mean ± Sd. All the statistical significance in this figure was determined using two-tailed Student's *t*-test by Microsoft Excel. “*” and “**” indicate a significant difference between transgenic *AaRVE1* plants and EV at *P* < 0.05 and *P* < 0.01, respectively.

To test whether heterologous expression of *AaRVE1* could accelerate the bud break in poplar, we performed the experiments under both greenhouse and field conditions. For the greenhouse experiments, the transgenic and WT plants were removed from field and transplanted in the greenhouse on December 26, 2019 and December 2, 2020, in each of 2 years. The L11 plants transplanted on December 26, 2019 sprouted in mid-January, 2020, ∼2 weeks earlier than WT. On February 4, 2020, the L11 plants formed fully expanded leaves while the WT were just breaking bud (Figure 2A). Similarly, both of the L6 and L11 plants transplanted in the greenhouse on December 2, 2020 showed earlier bud break compared with the EV plants (Figure 2B). Moreover, the L11, which has higher transgene *AaRVE1* expression, showed earlier bud break compared with the L6 (Figure 2, A and B).

In the field, the apical buds of L6 plants became more yellowish than WT (Figure 2C) in mid-April 2020. Note that the apical buds of L11 plants were killed by frost in the winter of 2019. Subsequently, the middle-upper buds of L11 plants became elongated, thin, and yellowish while the buds of WT and L6 were short and brownish red (Figure 2C). The buds of WT plants were darker brown compared with the L6 plants. In early May, the middle-upper buds of transgenic plants (L6 and L11) were more robust compared with the controls. The length of buds in L6 and L11 were substantially longer than WT, and the L11 buds were substantially longer than the L6 buds (Supplemental Figure 5). By June 20th, 2020, the WT plants and the L6 expressing *AaRVE1* plants formed normal apical bud on the main stem. Concurrently, the L11 lines, in which the terminal shoots were killed by low temperature in winter, formed three uniform and actively growing branches (Supplemental Figure 6). During the growing period from August 2019 to September 22nd, 2020, the height of WT and EV plants was increased by 7.2- and 6.7-fold, respectively, while the height of L11 and L6 plants was increased 7.1- and 6.8-fold, respectively, relative to their initial heights (Supplemental Figure 6). There were no significant differences (Statistical significance was determined using two-tailed Student's *t*-test. *P* < 0.05 was considered statistically significant.) between transgenic plants expressing *AaRVE1* and WT, probably due to the loss of dominance of the apical buds in L11 and its effects on growth.

In the year 2021, the buds of L6 showed earlier bud break compared with EV (Figure 2, D and E). Note that the upper buds and stems of L11 were again killed by freezing temperatures in the winter of 2020 (Supplemental Figure 7). Collectively, across both growing seasons, these results indicate that ectopic expression of *AaRVE1* substantially accelerated the bud break of poplar in spring under both greenhouse and field growth conditions.

### *AaRVE1* heterologous expression increases poplar biomass yield by 166% in the greenhouse

**Figure 3:**
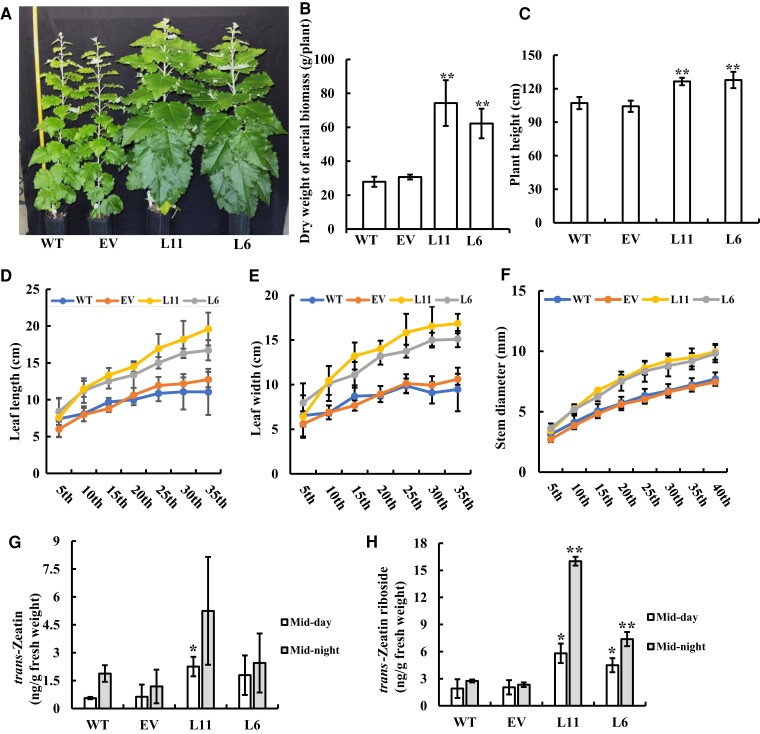
Phenotype characterization and hormone analysis of the transgenic *Populus* plants expressing *AaRVE1* in greenhouse. A, Phenotypes of around three-month-old transgenic poplar (L6, L11) expressing *AaRVE1* or EV, and wild-types (WT) under ORNL greenhouse conditions in winter. B, Dry weight of aerial biomass, (C) Plant height, (D) Leaf length, (E) Leaf width, and (F) Stem diameter of around five-month-old transgenic poplar grown in greenhouse in winter (*n* = 5). G, *trans*-Zeatin content and (H) *trans*-Zeatin riboside content in the young stem of transgenic plants and WT (*n* = 3). Data are presented as mean ± Sd. Statistical significance was determined using two-tailed Student's *t*-test. “*” and “**” indicates a significant difference between transgenic plants and WT at *P* < 0.05 and *P* < 0.01, respectively. All the statistical analysis in the figure were performed using the same method.

We grew the transgenic and WT plants during the winter season in a greenhouse (26.7°C and 16 h photoperiod with day length extended by supplemental lighting) at Oak Ridge National Laboratory in Tennessee. The L6 and L11 displayed faster growth in the greenhouse during the winter season, with an up to 1.66-fold increase in biomass yield and higher plant height, leaf length, leaf width and stem diameter in comparison with WT controls (Figure 3A–F).

### Heterologous expression of *AaRVE1* increases cytokinin contents

Phytohormones regulate plant growth, development, and dormancy processes. Cytokinin (CK) promotes the release of dormancy (Liu and Sherif, 2019). To explore the molecular mechanism underlying the dormancy regulation by *AaRVE1* in the poplar transgenic plants, we performed phytohormone profiling on the transgenic and WT plants grown in the greenhouse, and found that the contents of CKs (i.e. *trans*-Zeatin and *trans*-Zeatin riboside) in transgenic *AaRVE1* plants were significantly higher than those in WT plants (Figure 3, G and h), suggesting that *AaRVE1* regulates dormancy by increasing CK contents.

### *AaRVE1* represses multiple dormancy-related genes

**Figure 4:**
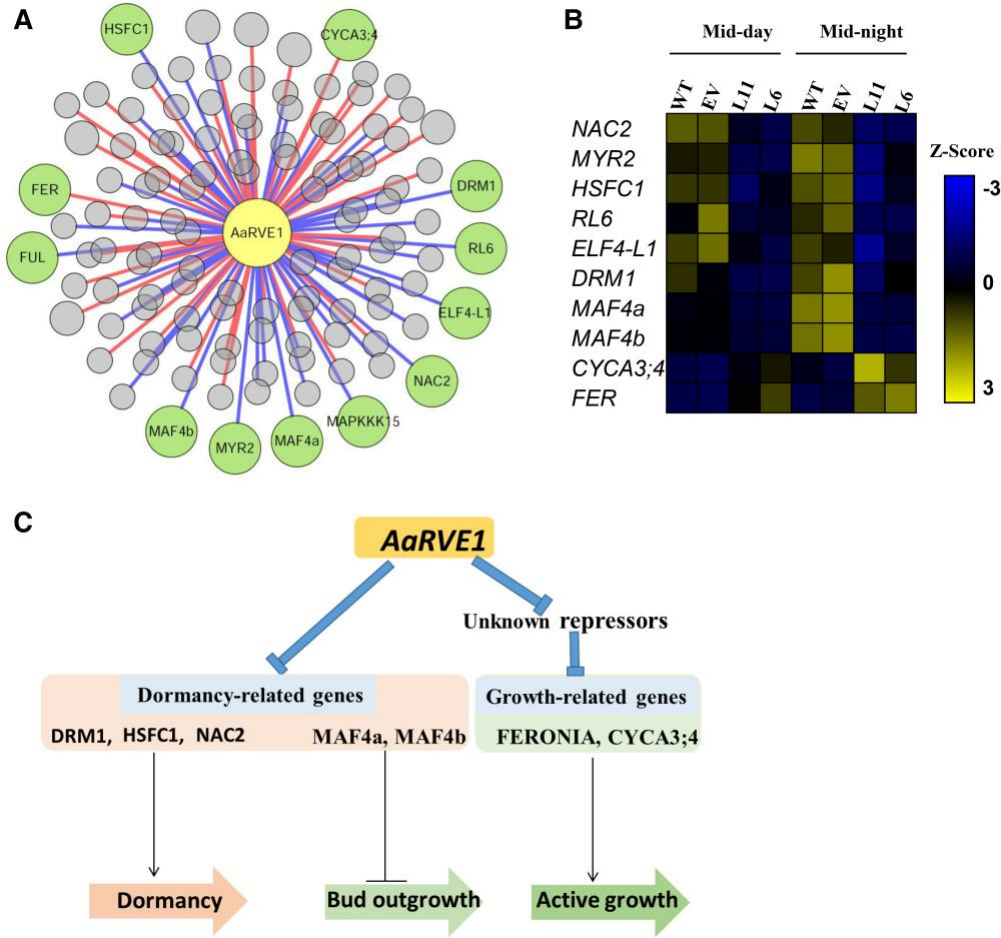
*AaRVE1* regulated multiple dormancy-related genes and cell division- and cell elongation-related genes. A, Co-expression network analysis of AaRVE1. The significantly DEGs identified by RNA-seq were subjected to co-expression analysis with the expression of the *AaRVE1* gene in the transgenic plants L6 and L11, EV, and wild-types (WT) (Supplemental Table 2). Green nodes represent the dormancy-related genes and bud break-related genes. Red and blue edges represent positive and negative co-expression relationships, respectively (*P* < 0.05 and |Correlation coefficient| > 0.7). B, Expression profiles of the dormancy-related genes and bud break-related genes at mid-day and mid-night. The gene information can be found in Supplemental Table 2. C, Diagram showing the regulation of bud dormancy and bud break in the transgenic plants expressing *AaRVE1*. DRM1: DORMANCY-ASSOCIATED PROTEIN 1; NAC2: NO APICAL MERISTEM FAMILY PROTEIN; HSFC1: HEAT SHOCK TRANSCRIPTION FACTOR C1; MAF: MADS AFFECTING FLOWERING (MAF)-like; CYCA3; 4: CYCLIN A3; 4.

To further explore the molecular mechanism underlying the delayed bud dormancy and accelerated bud break, we performed RNA sequencing (RNA-Seq) analysis of the transgenic lines and controls (Supplemental Table 1 and Supplemental Figure 8). Significantly differentially expressed genes (DEGs) were identified between transgenic plants and the controls (Supplemental Table 1). We found that 61 DEGs and 54 DEGs showed significant positive and negative correlation, respectively, with the *AaRVE1* expression (|Correlation coefficient| ≥ 0.7, *P* < 0.05; Figure 4A; Supplemental Table 2). The genes which have been shown to be related to bud dormancy and bud break were highlighted in Figure 4A–C. Other genes were summarized in Supplemental Table 2. *DORMANCY-ASSOCIATED PROTEIN 1* (*DRM1*) is a well-known “dormancy” marker in plant species (Stafstrom et al., 1998; González-Grandío et al., 2013). The expression level of *DRM1* was decreased in L11 and L6 in comparison with WT (Figure 4B). Furthermore, the transcript level of *DRM1* was significantly negatively correlated with the expression of *AaRVE1* (*r* = −0.755, *P* < 0.0001) in the transgenic poplar plants. NO APICAL MERISTEM FAMILY PROTEIN NAC2 and HEAT SHOCK TRANSCRIPTION FACTOR C1 (HSFC1) are associated with dormancy in plants (Nishitani et al., 2012; Tarancón et al., 2017). Here, we found that the transcript levels of *NAC2* and *HSFC1* were negatively correlated with *AaRVE1* with *r* = −0.871 (*P* < 0.0001) and *r* = −0.834 (*P* < 0.0001), respectively (Figure 4, A and B; Supplemental Table 2). Collectively, these findings indicate that the *AaRVE1* represses seasonal dormancy by repressing the expression of dormancy-related genes (Figure 4C).

MADS-domain protein FLOWERING LOCUS C (FLC)-like and its homolog MADS AFFECTING FLOWERING (MAF)-like emerged as promising candidates for playing roles in the repression of bud outgrowth during ecodormancy (Doğramacı et al., 2014; Porto et al., 2015). A recent study also suggests that *MdFLC-like* may prevent the outgrowth of dormant buds during the end of endodormancy and ecodormancy when winter temperature is low (Nishiyama et al., 2021). Here, we found that the transcription levels of *MAF4a* and *MAF4b* were significantly negatively correlated with the expression of *AaRVE1* (Figure 4, A and B; Supplemental Table 2). These findings suggest that the *AaRVE1* promote bud outgrowth by inhibiting the bud outgrowth repressors, such as *MAF4a* and *MAF4b* (Figure 4C).

### *AaRVE1* up-regulates bud break-related genes

Plant growth, including resumed growth after dormancy, consists of both cell division and cell elongation (Rohde and Bhalerao, 2007). Recently, it was reported that *FERONIA* promoted cell elongation (Deslauriers and Larsen, 2010; Liao et al., 2017) and *CYCLIN A3; 4* (*CYCA3; 4*) increased cell number in *Arabidopsis* (Willems et al., 2020). Here, we found that the expression levels of the *Populus FERONIA* and *CYCA3; 4* putative orthologs were positively correlated with the transcription level of *AaRVE1* (Figures 4A-B, Supplemental Table 2), suggesting that the *AaRVE1*-mediated promotion of bud break and active growth involves up-regulation of *FERONIA* and *CYCA3; 4* (Figure 4C).

### The MYB transcription factor *AaRVE1* functions as a transcriptional repressor

**Figure 5:**
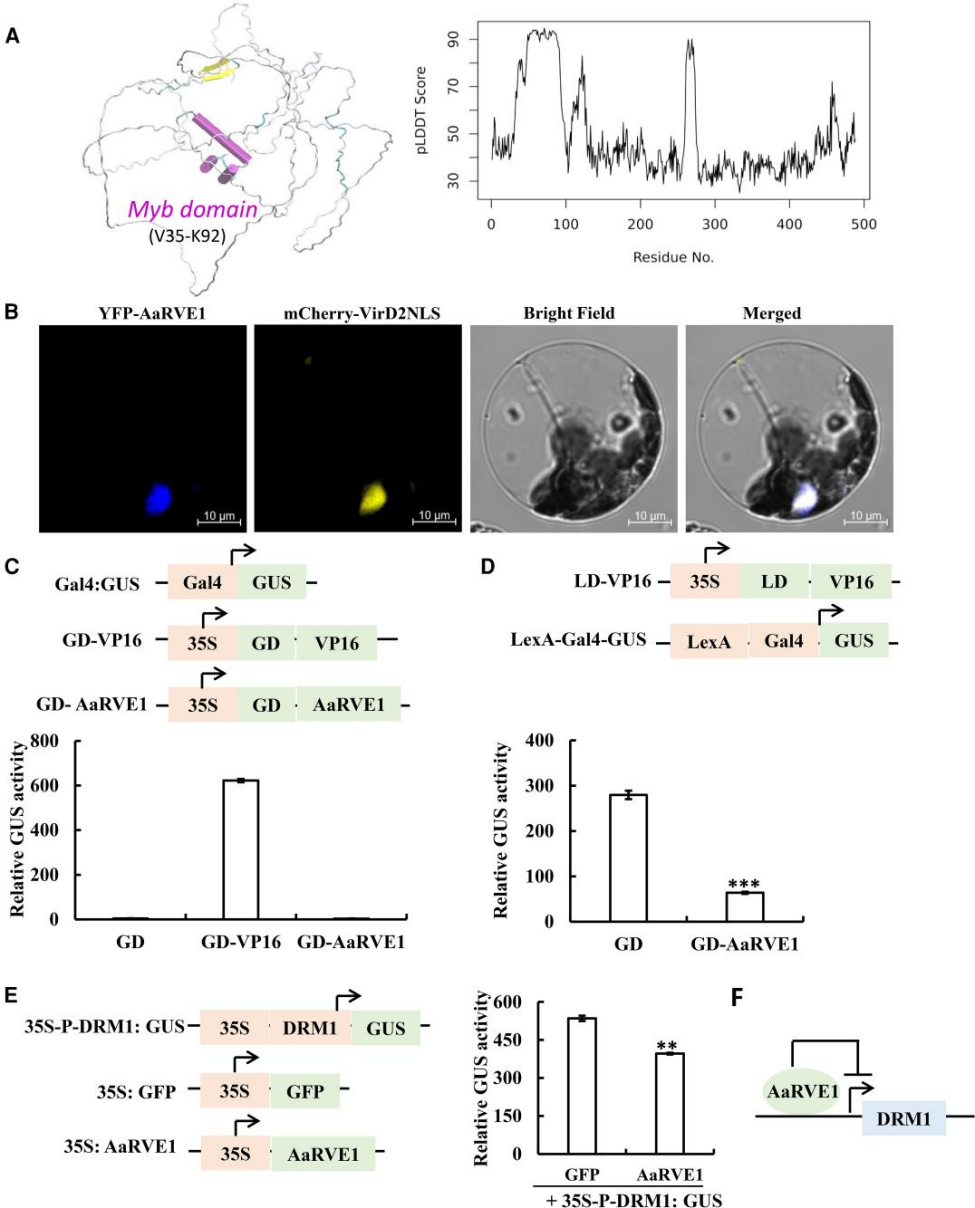
The MYB transcription factor *AaRVE1* functions as a transcription repressor and regulates the expression of *DORMANCY-ASSOCIATED PROTEIN 1* (*DRM1*) promoter. A, Protein structure modeling of AaRVE1 using AlphaFold2. AaRVE1 has a folded MYB domain surrounded by IDPRs. The predicted structure with the best overall predicted local distance differences test (pLDDT) score was shown. Purple cylinders are helices of the MYB domain. A beta-hairpin (yellow) is predicted with relatively high pLDDT scores. Except for the two folded regions (Myb domain and beta-hairpin), the rest part of the protein is disordered (white: coils; cyan: turns). B, AaRVE1 colocalizes with the nuclear marker VirD2NLS in *Populus* protoplasts. YFP: yellow fluorescent protein. C, AaRVE1 has no activator activity. AaRVE1 was fused with Gal4 binding domain (GD) and its activation on the reporter (Gal4:GUS) was measured in *Populus* protoplasts. GD only was used as the negative control. Transactivator GD-VP16 was used as the positive control. The 35S:Luciferase construct was co-transfected in each reaction to normalize and calculate the relative GUS activity. *n* = 3. D, AaRVE1 has repressor activity. The repression of GD-AaRVE1 on the reporter (activated LexA-Gal4:GUS by LD-VP16) was measured in protoplasts. For LD-VP16, the transcriptional activator VP16 was fused with LexA binding domain (LD). GD only was used as the negative control. 35S:Luciferase construct was co-transfected in each reaction to normalize and calculate the relative GUS activity. *n* = 3. E and F, AaRVE1 represses the expression of *DORMANCY-ASSOCIATED PROTEIN 1* (*DRM1*) promoter in protoplasts. In the reporter construct 35S-P-DRM1:GUS, the DRM1 promoter region was cloned downstream of the CaMV 35S promoter and upstream of the GUS reporter gene. The reporter construct 35S-P-DRM1:GUS was paired with the 35S:GFP (negative control) or 35S:AaRVE1 construct. The paired plasmids were co-transfected into *Populus* protoplasts. *n* = 3. Data are presented as mean ± Sd. Statistical significance was determined using two-tailed Student's *t*-test. “**” and “***” indicate a significant difference at *P* < 0.01 and *P* < 0.001, respectively. All the statistical analysis in the figure were performed using the same method.

To further provide insights into the regulation of dormancy-related genes by *AaRVE1*, we performed de novo structure prediction using AlphaFold 2 (AF2, V2.0.1) (Jumper et al., 2021). The AF2 structure indicate that the AaRVE1 protein has a MYB domain (V35 to K92) (Figure 5A; Supplemental Figure 9), consistent with the transcription factor function of RVE1 (Dubos et al., 2010). We also performed protein subcellular localization analysis and transcriptional activity assay of *AaRVE1* in *Populus* leaf mesophyll protoplasts. The *AaRVE1* fused to a yellow fluorescent protein (YFP) and a nuclear marker fused with mCherry tag (mCherry-VirD2NLS) were co-transfected into *Populus* protoplasts. The fluorescence signal of YFP-AaRVE1 fusion protein overlapped with VirD2NLS (Figure 5B), indicating that the AaRVE1 protein is localized in the nucleus, where it potentially regulates the transcriptions of its target genes. In the transcriptional activity assay, *AaRVE1* could not activate *GUS* reporter gene, while the positive control transcriptional activation domain VP16 (Cress and Triezenberg, 1991) robustly activated the *GUS* expression (Figure 5C). In contrast, *AaRVE1* significantly repressed the expression of *GUS* compared with the negative control (Figure 5D), demonstrating that *AaRVE1* function as a transcriptional repressor.

### AaRVE1 inhibits the activity of *DORMANCY-ASSOCIATED PROTEIN 1 (DRM1)* promoter

Given the transcript level of the well-known dormancy marker *DRM1* is significantly negatively correlated with the expression of *AaRVE1* (*r* = −0.755, *P* < 0.0001), we hypothesized that *AaRVE1* directly regulate *DRM1* promoter activity. To test this hypothesis, we cloned the *DRM1* promoter region into downstream of CaMV 35S promoter and upstream of *GUS* reporter gene (35S-P-DRM1:GUS). The reporter construct 35S-P-DRM1:GUS paired with 35S:GFP (negative control) or 35S:AaRVE1 construct were co-transfected into *Populus* protoplasts. As predicted, co-transfection of the 35S:AaRVE1 vector decreased GUS activity compared with that of 35S:GFP vector (Figure 5E), indicating AaRVE1 directly represses *DRM1* expression (Figure 5F).

### Sequence analysis and expression analysis of poplar *RVE* genes

Through BLAST analysis implemented in Phytozome (https://phytozome-next.jgi.doe.gov/blast-search), we found that the AaRVE1 protein (488 amino acids in length) had 39% (178/453) amino acid identity to rice RVE1-related gene (LOC_Os06g51260.1), 38% (184/484) amino acid identity to poplar RVE1-related gene (Potri.004G074300.1), and 46% (121/263) amino acid identity to *Arabidopsis* RVE1 (AT5G17300.1). Furthermore, our phylogenetic analysis revealed that the four RVE putative orthologs of poplar and four RVE putative orthologs of *Arabidopsis* belong to the same clade, and that three rice RVEs belongs another clade, while AaRVE1 falls into a separate clade (Supplemental Figure 10).

Transcript levels of the four *Populus RVE* genes at the beginning of dormancy when bud scales turn reddish brown in color and after the buds have flushed (fully open bud) were accessed using the gene expression data available in Phytozome (https://phytozome-next.jgi.doe.gov/info/Ptrichocarpa_v4_1; Supplemental Figure 11). The transcript levels of the poplar *RVE* genes (Potri.004G074300, Potri.017G144800 and Potri.012G038300) at the beginning of dormancy were lower than those after the buds have flushed. These data together with our *AaRVE1* overexpression results indicate that the *RVE* act as a regulator of bud dormancy and bud break in poplar. In addition, the transcript level of the “dormancy” marker gene *DRM1* at the beginning of dormancy was higher than that after the buds have flushed (Supplemental Figure 11), which is consistent with the role of *DRM1* in dormancy and consistent with our finding that *AaRVE1* represses the promoter activity of *DRM1*.

## Discussion

The bud dormancy in the fall and bud break in the spring in woody perennial plants were thought to be regulated by different molecular mechanisms (Ding and Nilsson, 2016). A few genes responsible for the onset of bud dormancy have been identified in trees, such as *CONSTANS* (*CO*)/*FLOWERING LOCUS T* (*FT*) regulatory module (Böhlenius et al., 2006), abscisic acid signaling gene *ABI1* (Tylewicz et al., 2018) and *DRM1* (Stafstrom et al. 1998; González-Grandío et al. 2013). Several genes have been shown to be involved in bud break, such as *CENTRORADIALIS 1* (*CEN1*) (Mohamed et al., 2010), *EARLY BUD BREAK 1* (*EBB1*) (Yordanov et al., 2014), and *SHORT VEGETATIVE PHASE-LIKE* (*SVL*) and its downstream target *TCP18* (Singh et al., 2018). To date a single molecular element or master regulator that delays the onset of bud dormancy in the fall and advances bud break in the spring in deciduous trees has not been discovered. In this study, we transferred the single *AaRVE1* gene into *Populus* and found that heterologous expression of *AaRVE1* not only delayed the onset of bud dormancy but also accelerated the bud break in the field (Figures 1 and 2). Our transcriptome analysis revealed that the expression levels of multiple dormancy-related genes were decreased while the expression levels of bud break-related genes were increased in the transgenic plants expressing *AaRVE1* (Figure 4). Furthermore, we found that AaRVE1 protein was localized in the nucleus, with a capability to represses the activity of *DRM1* promoter (Figure 5). Our results indicate that *AaRVE1* functions as a regulator of bud dormancy and bud break.

*RVE1* belongs to a small subfamily of the MYB-like transcription factor family. There are two reports on the *RVE* transcription level changes around the time of dormancy release in sweet cherry (Vimont et al., 2019) and during the chilling treatment, which is required for bud break of apple trees (Porto et al., 2015). However, the function of *RVE* in tree dormancy and bud break has not been determined through molecular genetics approaches (e.g. overexpression) yet. Also, although *DRM1* is a well-known dormancy marker gene, its regulator remains elusive. Here, we created the transgenic poplar plants expressing *AaRVE1* and demonstrated that *AaRVE1* was involved in bud dormancy and bud break through phenotype characterization, hormone analysis and transcriptome analysis (Figures 1–4). Furthermore, we revealed that *AaRVE1* had a folded MYB domain and demonstrated that *AaRVE1* could repress the activity of *DRM1* promoter (Figure 5). Finally, we found that the transcript levels of the poplar *RVE* genes (Potri.004G074300, Potri.017G144800, and Potri.012G038300) at the beginning of dormancy were lower than that after the buds have flushed. The transcript level of *DRM1* in poplar at the beginning of dormancy was higher than that after the buds have flushed (Supplemental Figure 11). The expression level of *DRM1* in Kiwifruit is inversely associated with spring budbreak (Wood et al., 2013). Collectively, these results provide insights into the molecular mechanisms underlying the *RVE-DRM1* regulatory module involved in tree dormancy and bud break.

The transgenic plants expressing *AaRVE1* exhibited delayed onset of bud dormancy in the fall (Figure 1), which lead to freeze damage and death of the apical buds during the winter in the open field in Storrs, CT (41.8048° N, 72.2930° W) (Figure 1; Supplemental Figure 7) and loss of dominance of the apical buds in the next year (Supplemental Figure 6). The results suggest that *AaRVE1* could be potentially employed to develop “evergrowing” trees for frost-free temperature or subtropical areas.

In conclusion, we demonstrate that the *AaRVE1* functions as a regulator of bud dormancy and bud break by repressing multiple dormancy-related genes and up-regulating bud break-related genes. These findings have important implications for developing “evergrowing” trees for frost-free areas as a means to increase crop yield and enhance carbon sequestration for climate change mitigation.

## Materials and methods

### Generation of transgenic plants

The coding sequence of *AaRVE1* (Aam022373) (Yin et al., 2018) fused to two FLAG epitope tags (Terpe, 2003) was chemically synthesized by Integrated DNA Technology (Coralville, IA) and used to produce a chimeric gene construct, p35S:*FLAG-AaRVE1*/pNOS: *nptII*. The vector contained the CaMV35S promoter driving *FLAG-AaRVE1* and nopaline synthase (NOS) promoter driving the *nptII* gene for kanamycin resistance as a selection marker. The vector and empty vector (EV) pBI121 was delivered into the *Agrobacterium tumefaciens* strain, GV3101, using the freeze–thaw method (Höfgen and Willmitzer, 1988) for plant transformation, respectively.

Poplar (*P. tremula* × *P. alba* clone 717-1B4) was used for genetic transformation. The generation and culturing of transgenic plants were carried out as previously described (Meilan and Ma, 2006). Leaves and petioles from in vitro-grown plants were used for *Agrobacterium*-mediated transformation. Explant preparation and pre-culture, inoculation with *Agrobacterium* and co-cultivation, callus induction and shoot regeneration, and rooting regenerants were performed according to an *Agrobacterium*-mediated poplar transformation protocol developed by Meilan and Ma (2006).

The expression level of *AaRVE1* gene in the wild-type and transgenic plants were analyzed by reverse transcription quantitative PCR (RT-qPCR) as previously described (Liu et al., 2021). *Actin* gene was used as an internal control. Primers were provided in Supplemental Table 3.

### Field evaluations of transgenic plants

Transgenic, WT, and EV plants with similar plant heights were grown in the greenhouse and acclimated outdoors for two weeks before transplanted into field on August 15, 2019 and July 28, 2020. Plants were placed in a completely random design layout (1.5 m × 1.5 m spacing) at the University of Connecticut Depot Campus, Storrs, CT (41.8048° N, 72.2930° W). Plant height, stem diameter, bud and shoot length, bud dormancy, and bud break were recorded. Data are presented as mean ± Sd. Statistical significance was determined using two-tailed Student's *t*-test by Microsoft Excel.

### Phytohormones profiling

Wild-type plants and transgenic plants were planted under 16-h-light/8-h-dark at ORNL greenhouse. A total of 24 young stem samples were collected with three biological replicates sampled at mid-day and mid-night. The samples were frozen in liquid nitrogen and ground to a fine powder and stored at −80°C. Phytohormone analysis was carried out by Proteomics & Mass Spectrometry Facility at the Danforth Plant Science Center.

### Transcriptome sequencing

For total RNA isolation, approximately 100 mg cryogenically frozen and ground tissue was added to 850 μl of CTAB buffer with 1.0% (v/v) β-Mercaptoethanol. The samples were incubated while shaking at 56°C for 5 min after which 600 μl chloroform:isoamylalcohol (24:1) was added and centrifuged at full-speed for 8 min at room temperature. The clear supernatant was carefully transferred into a filter column provided in the Spectrum Plant Total RNA Kit (Sigma, Cat. No. STRN250-1KT) and centrifuged for 1 min at full-speed to remove plant tissue debris. The remaining steps were performed with the Spectrum Plant Total RNA Kit according to the manufacturer's instructions. An on-column DNase treatment was applied to remove residual genomic DNA contamination. The final RNAs were eluted with 50 μl of RNase free water from the binding columns and RNA quantity and quality were examined using a NanoDrop 1000 spectrophotometer (Thermo Scientific, Wilmington, DE, USA).

PerkinElmer Sciclone NGS robotic liquid-handling system was used to perform plate-based RNA sample prep using Illumina's TruSeq-Stranded mRNA HT sample prep kit utilizing poly-A selection of mRNA following the Illumina protocol. The conditions are: 1 µg of sample total RNA starting material per sample and 8 PCR cycles was used for library amplification. Roche LightCycler 480 real-time PCR instrument and KAPA Biosystem's next-generation sequencing library qPCR kit were used to quantify the prepared libraries. Then the quantified libraries were multiplexed and were sequenced on the Illumina NovaSeq 6000 sequencing platform with the reagent kits NovaSeq XP v1.0, S4 flow cell, following a 2 × 150 indexed run recipe.

### RNA-seq reads mapping and data analysis

JGI QC pipeline was used to filter and trim Raw fastq file reads. Raw reads from each library were aligned to the *P. trichocarpa* reference genome (https://phytozome.jgi.doe.gov) using TopHat2 (Kim et al., 2013). The reads which mapped uniquely to one locus were counted. The raw gene counts, which were used to evaluate the correlation level between biological replicates, was generated using FeatureCounts (Liao et al., 2014). DEGs between pairs of conditions (*P* < 0.05) were determined using DESeq2 (Love et al., 2014).

GO enrichment was performed using agriGO (Tian et al., 2017). For co-expression network construction, Pearson correlations were calculated in parallel between all pairs of gene expression vectors. The threshold |Correlation coefficient| ≥ 0.7 (*P* < 0.05) was applied to resulting correlations and the remaining correlations were visualized by Cytoscape 3.7.0 (Shannon et al., 2003).

### Protein structure modeling

AlphaFold 2 (AF2, V2.0.1) (Jumper et al., 2021) was used for protein structure predictions. The predicted local distance differences test (pLDDT) score serves as the “confidence” scores in the AF2 predictions, whereas the residues with pLDDT larger than 70 are considered as high confidence in the prediction, the residues with low pLDDT (e.g. < 50) are often in the intrinsically disordered protein regions (IDPRs) (Ruff and Pappu, 2021; Akdel et al., 2022).

### Subcellular localization in *Populus* protoplasts

The subcellular localization of AaRVE1 was analyzed in *Populus* mesophyll protoplasts as previously described (Xie et al., 2018). In brief, the *YFP* fusion *AaRVE1* driven by CaMV 35S promoter construct (8 µg) were co-transfected with nuclear marker VirD2NLS-mCherry construct (2 µg) into 100 μl of protoplasts. After 12–14 h incubation under weak light, mCherry fluorescence and YFP fluorescence were examined and photographed. Zeiss LSM 710 confocal microscope was used to collect the images. YFP: Laser 488 nm, detection wavelength 500–530 nm, detector gain 800. mCherry: Laser 561 nm, detection wavelength 565–615 nm, detector gain 750. Zeiss ZEN software package was used to process the images.

### Transcriptional activity assay

Transcriptional activity of AaRVE1 was tested in *Populus* mesophyll protoplasts as previously described (Xie et al., 2018). For activator activity assays, the reporter construct Gal4:GUS was paired with 35S:Gal4 DNA Binding Domain (GD, negative control), 35S:GD-VP16 (positive control), or 35S:GD-AaRVE1 construct. For repressor activity assays, the reporter construct LexA-Gal4:GUS together with the transactivator construct 35S: LexA DNA Binding Domain-VP (35S:LD-VP) were paired with 35S:GD (negative control) or 35S:GD-AaRVE1 construct. For the repression of *DRM1* promoter, the *DRM1* promoter sequence was chemically synthesized by Integrated DNA Technology (Coralville, IA, USA) and used to produce a reporter construct 35S-P-DRM1:GUS. The reporter construct 35S-P-DRM1:GUS was paired with 35S:GFP (negative control) or 35S:AaRVE1 construct. A total of 10 µg of paired plasmids was co-transfected into 100 μl of protoplasts and then incubated for 18–20 h in dark. In total, 100 ng of 35S:Luciferase plasmid was co-transfected in each transfection as an internal control. GUS and luciferase activities were measured to calculate the relative GUS activity (GUS/LUC). Three technical replicates were performed to calculate mean values and standard deviations. Experiments were repeated for three times with consistent result.

### Phylogenetic analysis

Phylogenetic tree of AaRVE1 (Aam022373) (Yin et al., 2018) and the RVEs from poplar, Arabidopsis, and rice were made using IQ-tree (http://iqtree.cibiv.univie.ac.at/). The tree was rooted on Midpoint. Scale bar = 0.5 substitutions per site. The accession numbers of RVEs from poplar, Arabidopsis and rice are provided in Supplemental Figure 10.

### Accession numbers

The accession numbers of poplar genes/proteins mentioned in the paper are provided in either Supplemental Table 2 or Supplemental Figure 10. The sequences are available in Phytozome (https://phytozome-next.jgi.doe.gov/). The accession numbers of *RVEs* of Arabidopsis and rice are provided in Supplemental Figure 10.

## Supplementary Material

kiac588_Supplementary_Data

## Data Availability

The RNA-seq data were deposited in NCBI SRA (SRP197699- SRP197704, SRP197714-SRP197719, SRP197721-SRP197728, SRP197730, SRP197731, SRP197734, and SRP197743).

## Supplemental data

The following materials are available in the online version of this article.

**Supplemental Figure S1.** Schematic representation of the T-DNA vector for the heterologous expression of *AaRVE1* in *Populus*.

**Supplemental Figure S2.** Field trial of the transgenic plants in Connecticut.

**Supplemental Figure S3.** Phenotype characterization of the transgenic plants in field.

**Supplemental Figure S4.** Phenotype characterization of the transgenic plants in the year 2020 in field.

**Supplemental Figure S5.** Heterologous expression of *AaRVE1* accelerated bud break in the field.

**Supplemental Figure S6.** Phenotype characterization of the transgenic plants which were planted in August 2019 over approximately one year.

**Supplemental Figure S7.** The upper buds and stems of L11 were frozen to death in the winter of 2020.

**Supplemental Figure S8.** Gene ontology enrichment analysis of differentially expressed genes in the biological process category (P), molecular function category (F) and cellular component category (C).

**Supplemental Figure S9.** The predicted aligned error (PAE) map of RVE1 indicates that the MYB domain (dark green) is well-folded.

**Supplemental Figure S10.** Phylogenetic relationships of *AaRVE1* (Aam022373) and its putative orthologs from poplar, Arabidopsis, and rice.

**Supplemental Figure S11.** Transcript level of *Populus RVE* and *DRM1* genes at the beginning of dormancy when bud scales turn reddish brown in color (early dormant bud) and after the buds have flushed (fully open bud).

**Supplemental Table S1.** Differentially expressed genes between pairs of conditions (*P* < 0.05).

**Supplemental Table S2.** Genes co-expressed with *AaRVE1*.

**Supplemental Table S3.** Primers used in this study.
