# Supplementary material for: Agave REVEILLE1 regulates the onset and release of seasonal dormancy in Populus
Source: Plant Physiol. 2022 Dec 22;191(3):1492–504. doi: 10.1093/plphys/kiac588 (PMC10022617; doi:10.1093/plphys/kiac588)
Supplement: kiac588_Supplementary_Data [file kiac588_supplementary_data.zip › AaRVE1_Supplemental Figures_PP_Final_R1_20221218.pdf]

Supplementary Figure 1

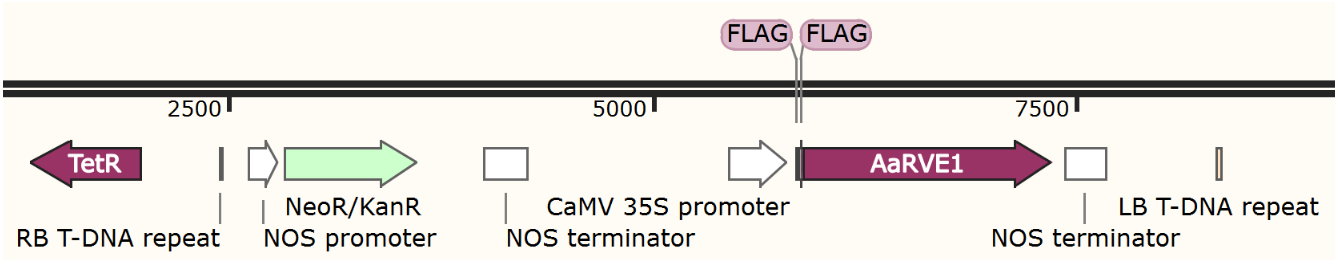

Supplemental Figure S1. Schematic representation of the T-DNA vector for the heterologous expression of *AaRVE1* in *Populus*. The coding sequence of *AaRVE1* (Aam022373) was fused to two FLAG epitope tags. Cauliflower mosaic virus 35S (CaMV35S) promoter was used to drive the FLAG-AaRVE1. FLAG: epitope tag. LB and RB: the left and right border of T-DNA, respectively. NOS: nopaline synthase; KanR: kanamycin resistance gene.

Supplementary Figure 2

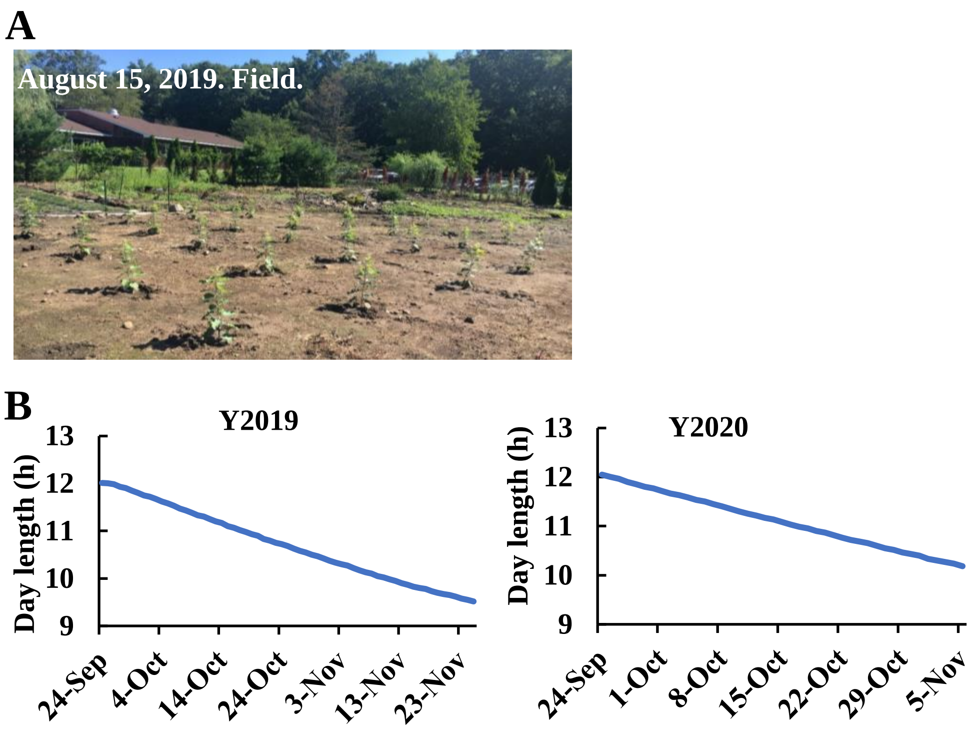

Supplemental Figure S2. Field trial of the transgenic plants in Connecticut. (**A**) Overview of the field trial. (**B**) Day length in the field of Storrs, CT.

Supplementary Figure 3

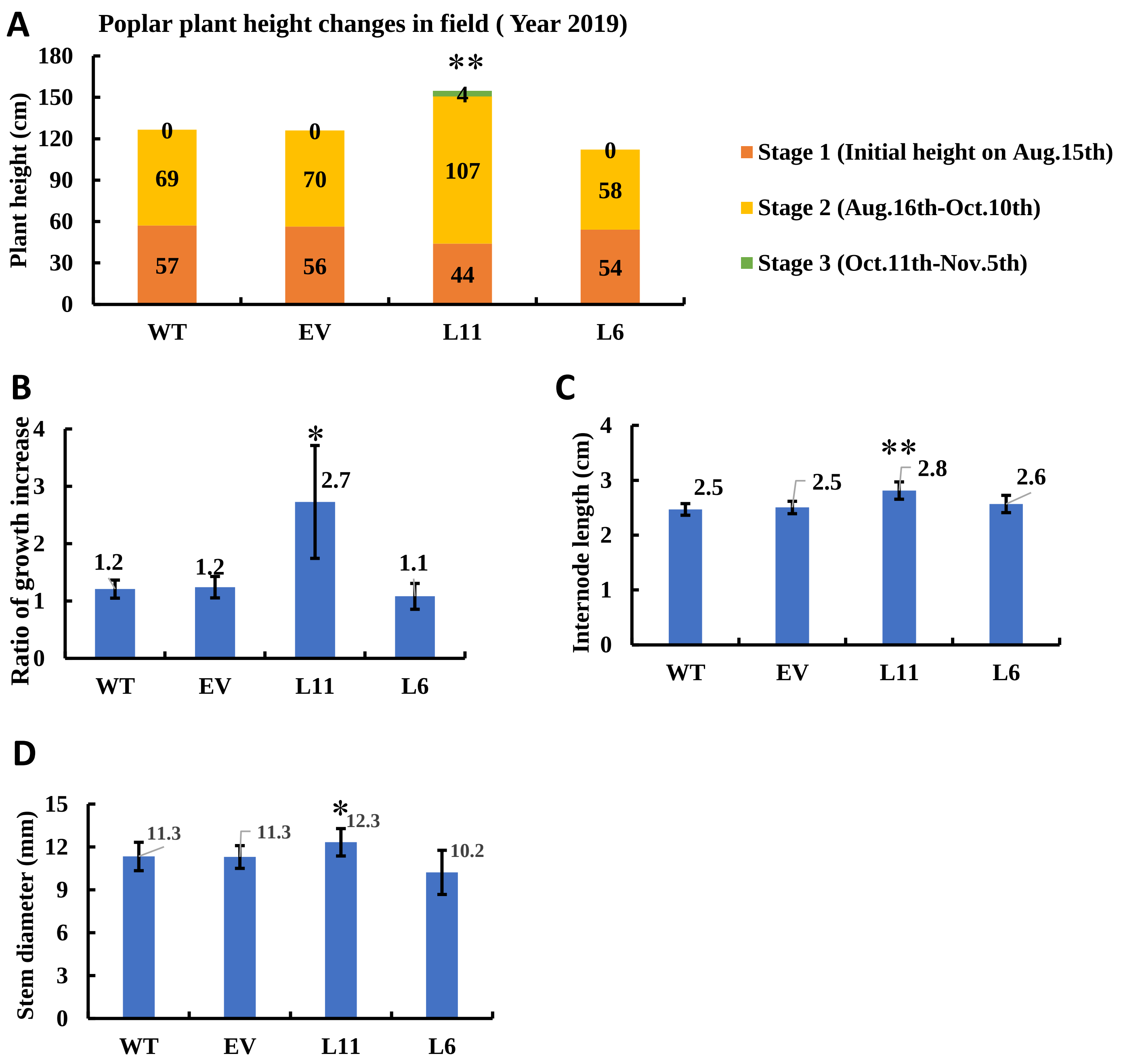

Supplemental Figure S3. Phenotype characterization of the transgenic plants in field. (**A**) Plant height of transgenic poplar (L6, L11) expressing *AaRVE1* or empty vector (EV), and wild types (WT) in the field of Storrs, CT. (**B**) The ratio of growth increase. Internode length (**C**) and stem diameter (**D**) of the transgenic plants. Data is presented as mean ±SD. Statistical significance was determined using two-tailed Student's t-test of Microsoft Excel. "*" and "**" indicate a significant difference between transgenic plants and WT at P<0.05 and P<0.01, respectively. WT, n=8; EV, n=9; L11, n=6; L6, n=9.   All the statistical analysis in the figure were performed using the same method.

Supplementary Figure 4

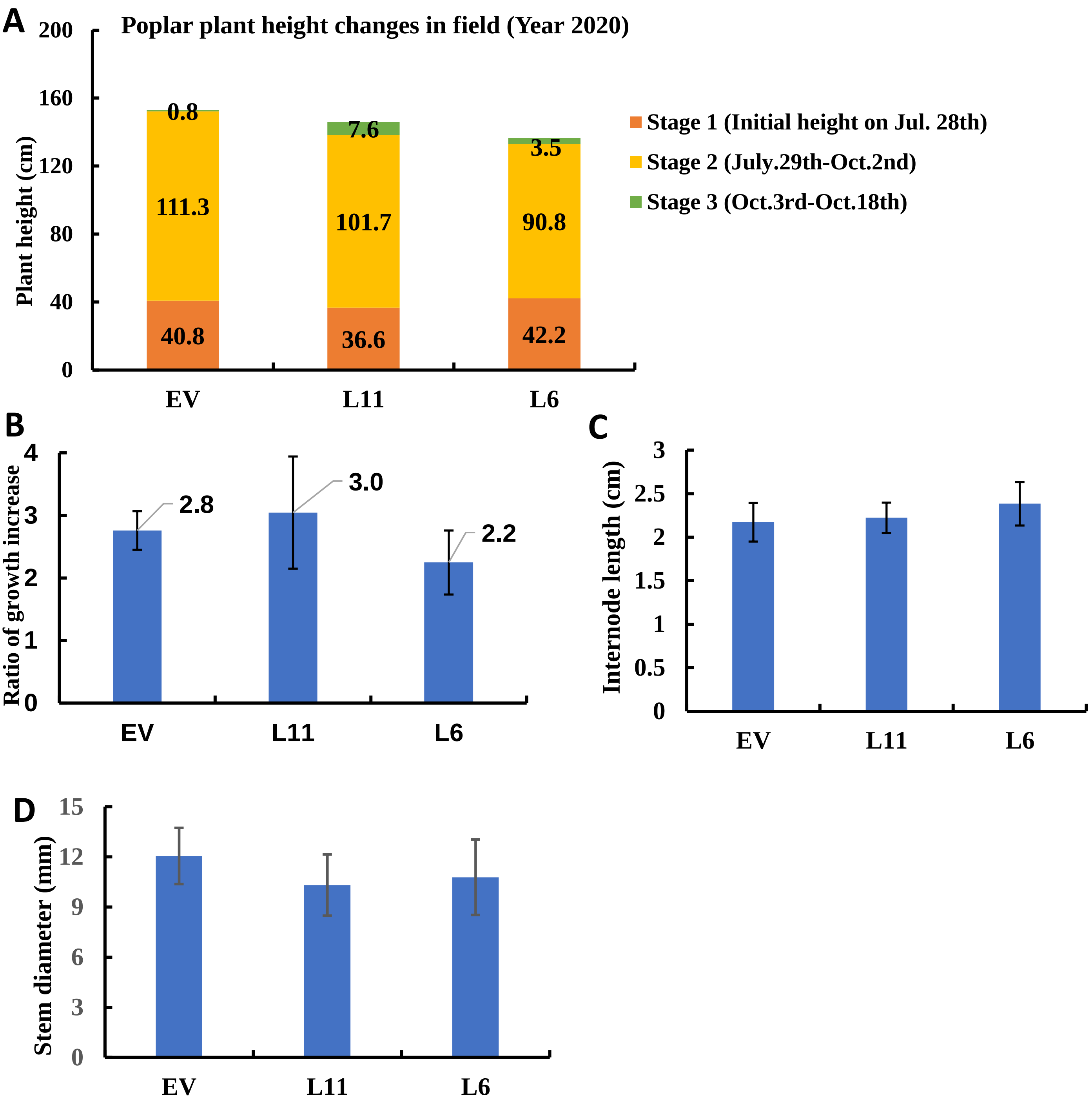

Supplemental Figure S4. Phenotype characterization of the transgenic plants in the year 2020 in field. (**A**) Plant height of transgenic poplar (L6, L11) expressing *AaRVE1* or empty vector (EV) in the field of Storrs, CT. (**B**) The ratio of growth increase. Internode length (**C**) and stem diameter (**D**) of the transgenic plants. All the data in the figure is presented as mean ±SD. EV, n=6; L6, n=7; L11, n=6.

Supplementary Figure 5

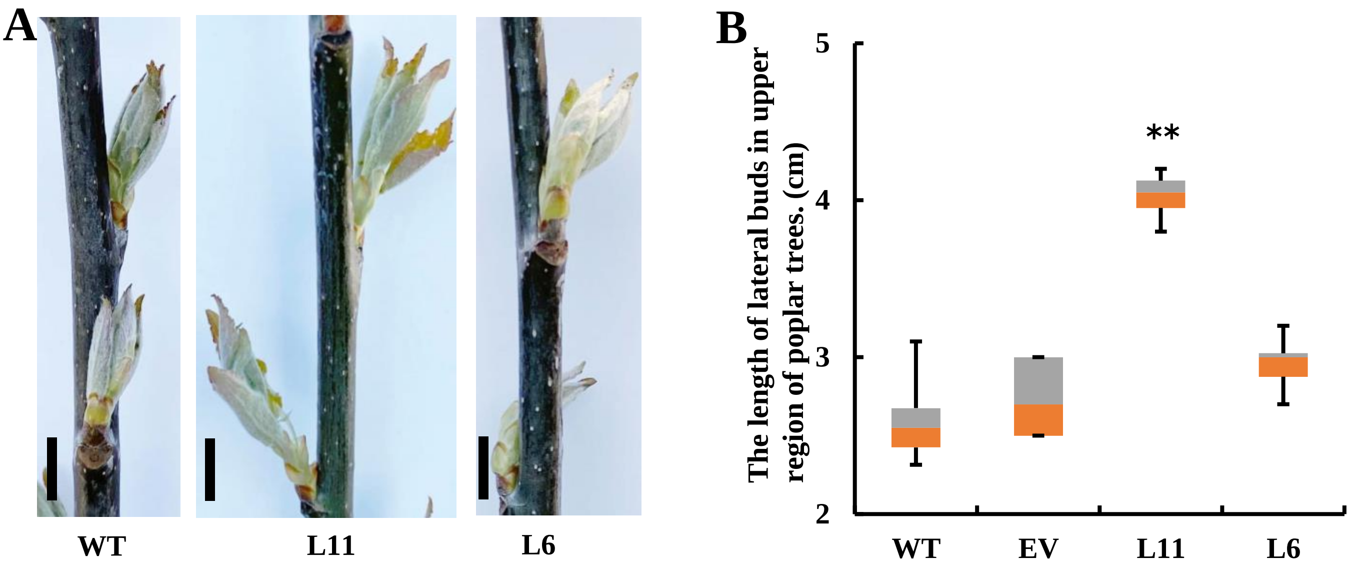

Supplemental Figure S5. Heterologous expression of *AaRVE1* accelerated bud break in the field. (**A**) Poplar buds in middle-upper part of poplar grown in filed. Photos were taken on May 5, 2020. Bars=1 cm. (**B**) The length of lateral buds in the upper part of poplars grown in field. Data were collected on May 5, 2020. In each box, the lower and upper boundaries represent the 25th and 75th percentiles, respectively. The bottom edge of gray block (the top edge of orange block) represents the median. The whiskers represent 1.5× the interquartile range. WT, n=6; EV, n=3; L6, n=8; L11, n=4. Statistical significance was determined using two-tailed Student's t-test of Microsoft Excel. “**” indicates a significant difference between transgenic plants and WT at $P<0.01$.

Supplementary Figure 6

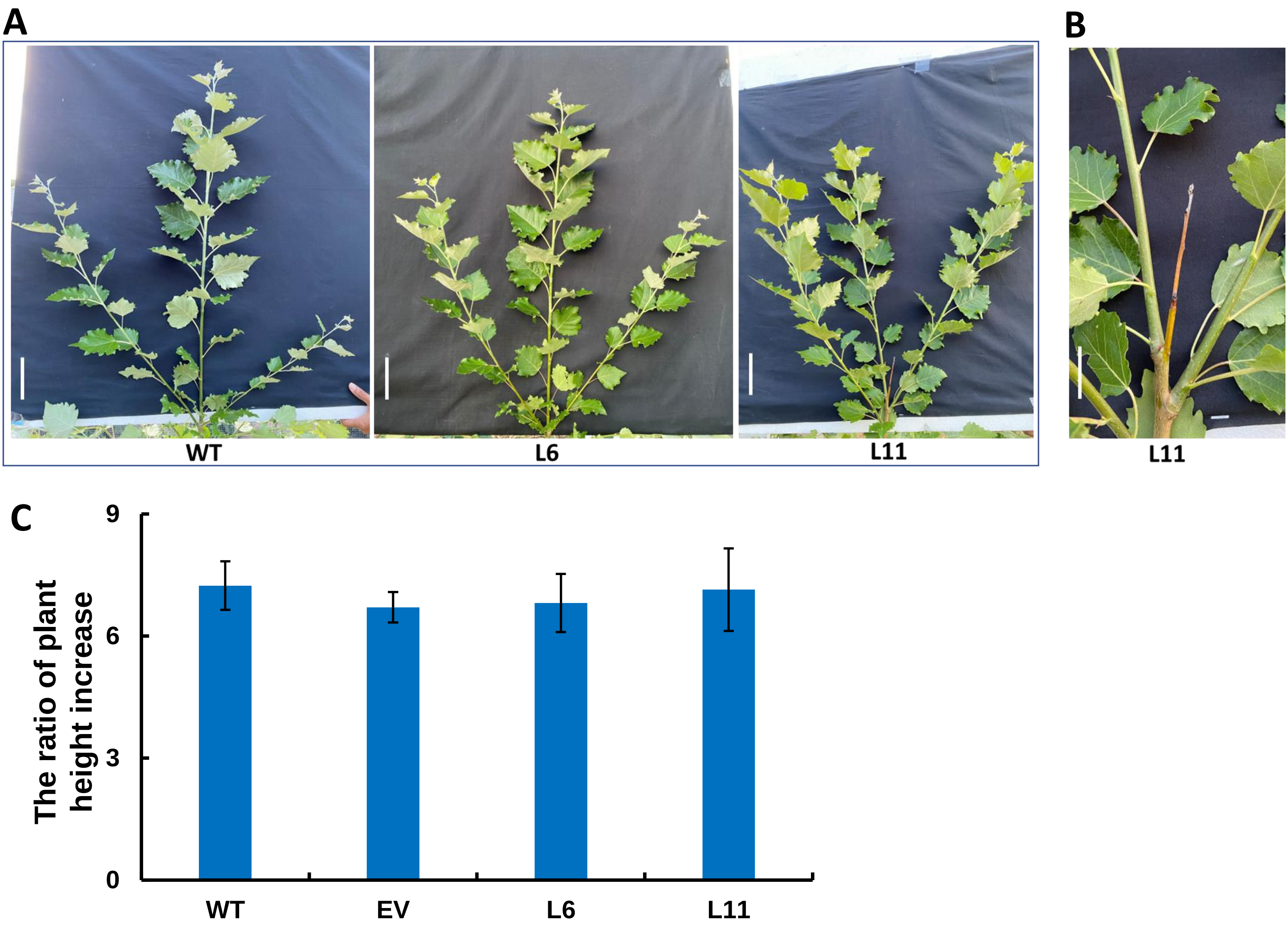

Supplemental Figure S6. Phenotype characterization of the transgenic plants which were planted in August 2019 over approximately one year. (**A-B**) The growth of branches on the top of poplars in the field of Storrs, CT. The normally dormant plants (wild type (WT) and L6 expressing AaRVE1) that have normal apical buds formed 1 main branch and 2 medium size branches, L11 lines expressing AaRVE1, the terminal shoots of which were killed by low temperature (**B**), formed 3 uniform and medium growing branches (**A**). Photos were taken on Jun. 20th, 2020. For A, Bar=10 cm. For B, Bar=3 cm. (**C**) The ratio of plant height increase in September 2020 compared to August 2019. No significant differences were observed between transgenic plants expressing AaRVE1 and WT. EV: empty vector. Data is presented as mean ±SD (WT, n=6; EV, n=3, L6, n=8; L11, n=4). Statistical significance was determined using two-tailed Student's t-test of Microsoft Excel. $P<0.05$ was considered statistically significant.

Supplementary Figure 7

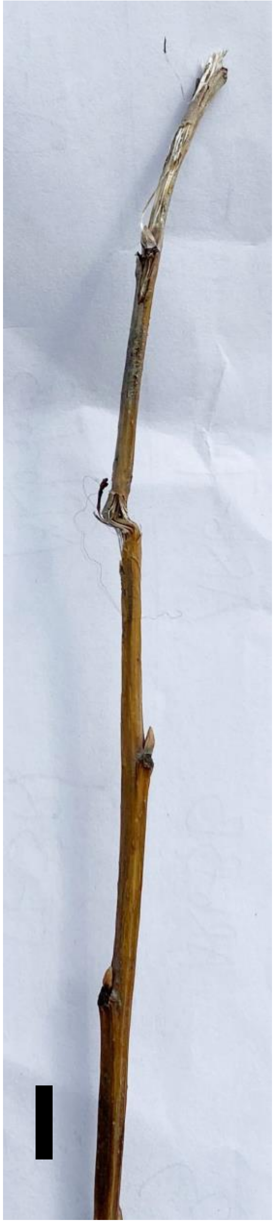

Supplemental Figure S7. The upper buds and stems of L11 were frozen to death in the winter of 2020. This photo was taken on April 20, 2021. Bar= 1 cm. L11: transgenic plants expressing *AaRVE1*.

Supplementary Figure 8

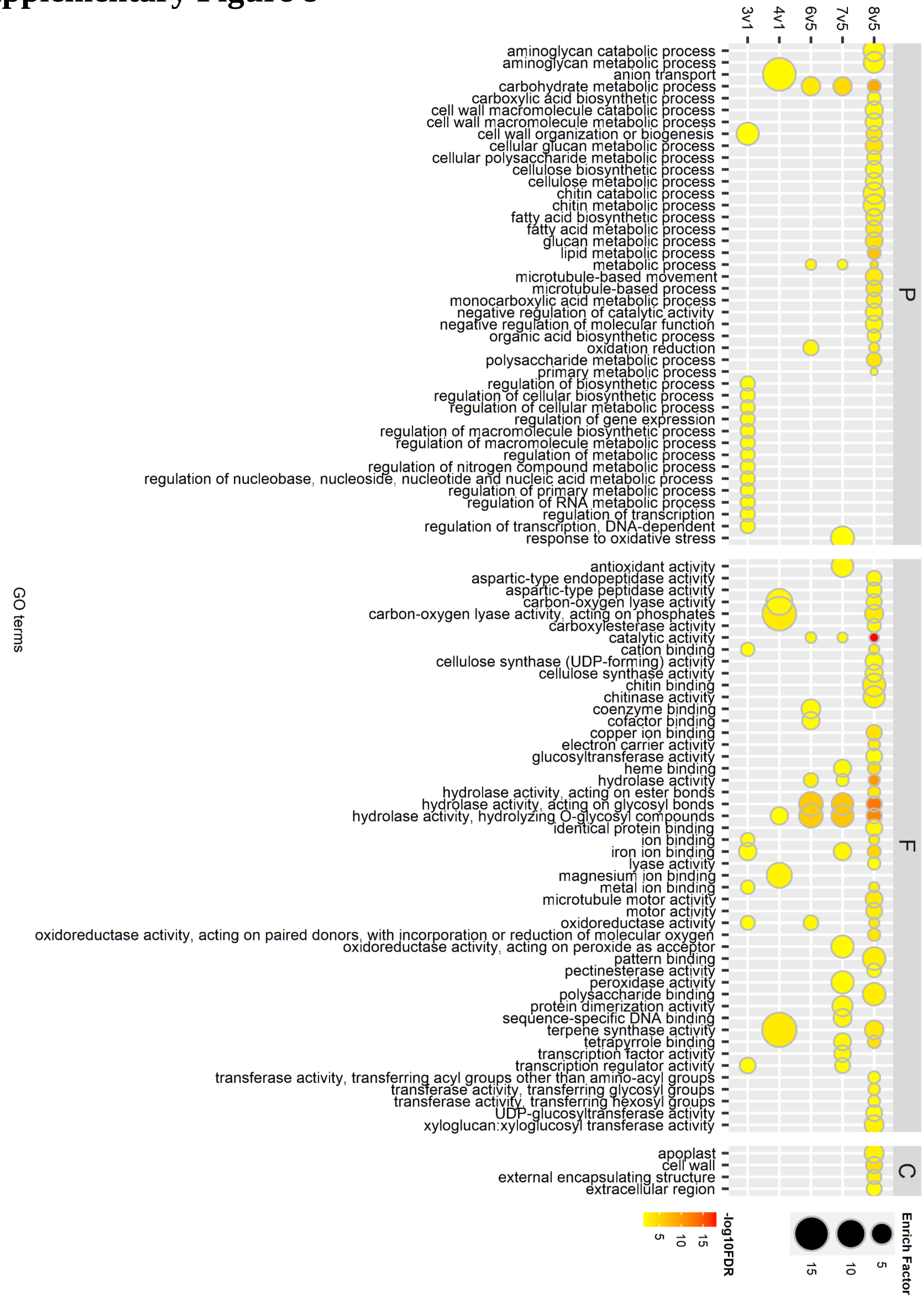

Supplemental Figure S8. Gene ontology enrichment analysis of differentially expressed genes in the biological process category (P), molecular function category (F) and cellular component category (C). Definitions of 1-8 are in Supplemental Table S1.

Supplementary Figure 9

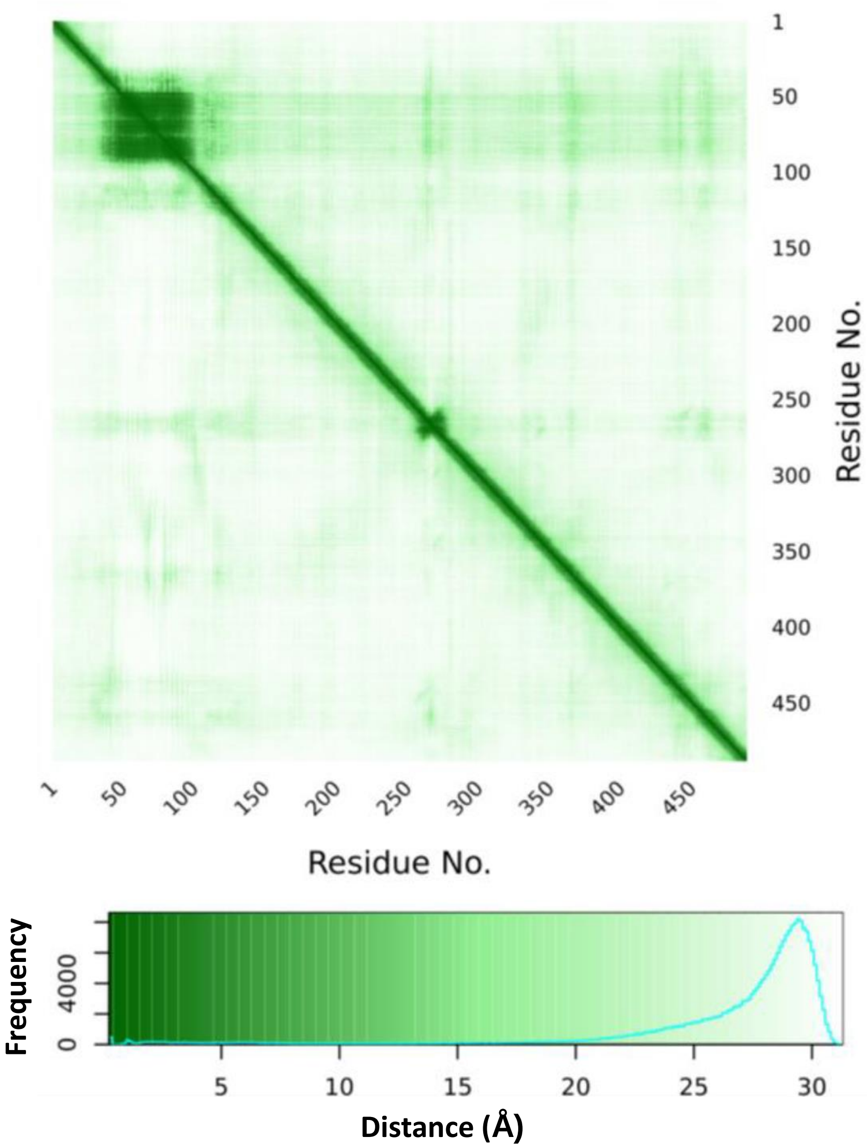

Supplemental Figure S9. The predicted aligned error (PAE) map of RVE1 indicates that the MYB domain (dark green) is well-folded.

Supplementary Figure 10

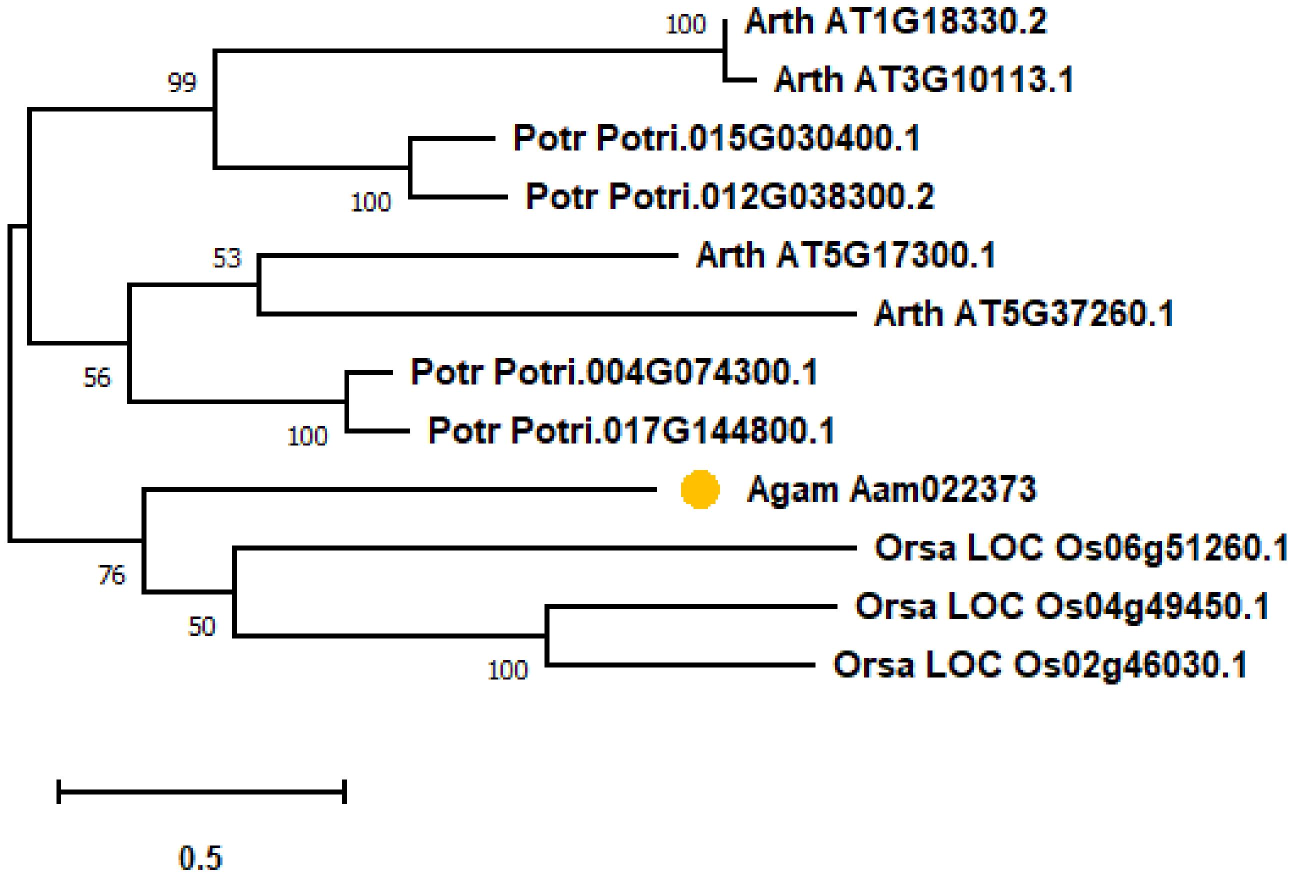

Supplemental Figure S10. Phylogenetic relationships of AaRVE1 (Aam022373) and its putative orthologs from poplar, Arabidopsis, and rice. The phylogenetic tree was made using IQ-tree (http://iqtree.cibiv.univie.ac.at/). The tree was rooted on Midpoint. Scale bar = 0.5 substitutions per site.

Supplementary Figure 11

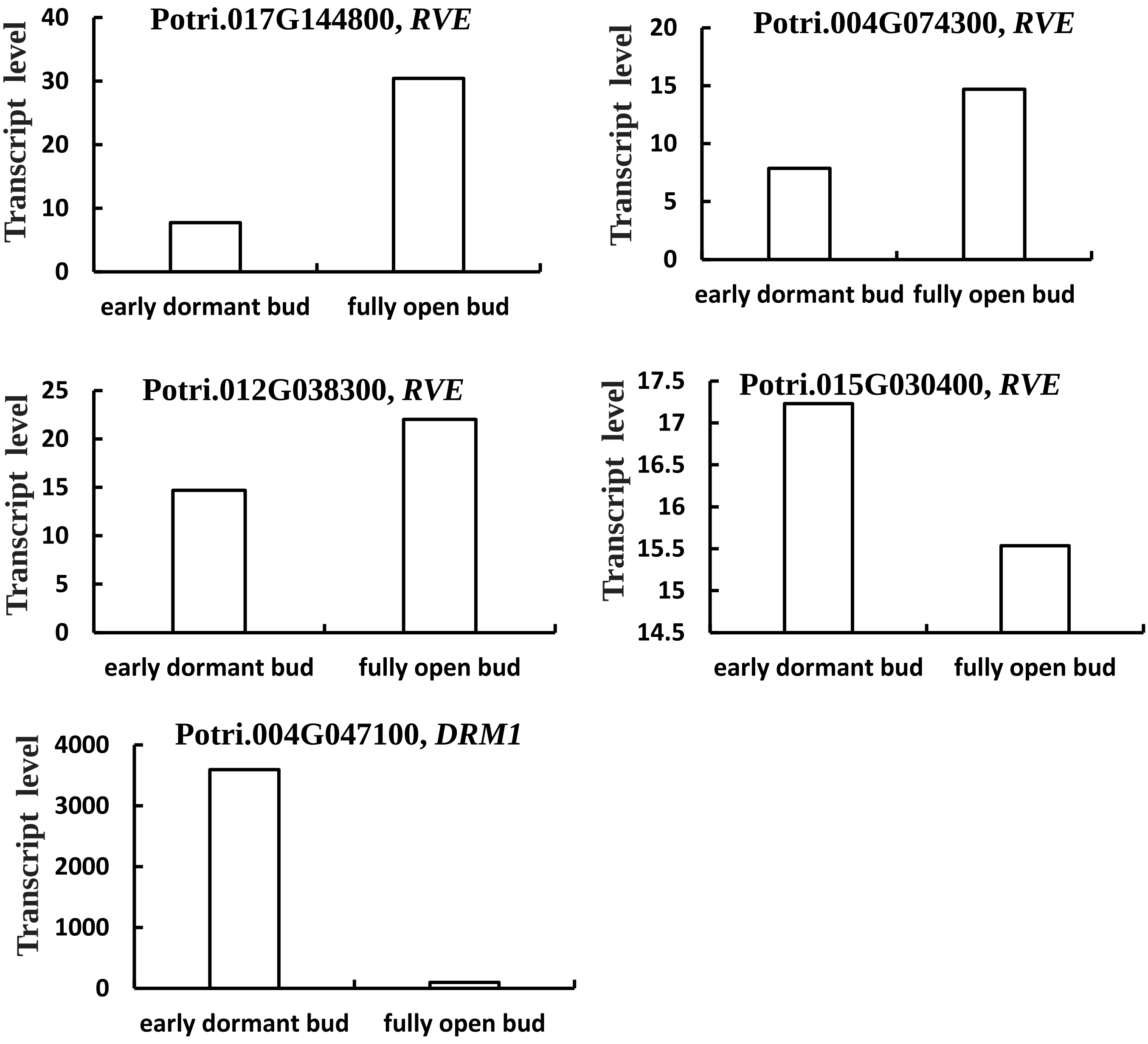

Supplemental Figure S11. Transcript level of *Populus RVE* and *DRM1* genes at the beginning of dormancy when bud scales turn reddish brown in color (early dormant bud) and after the buds have flushed (fully open bud). RNA-seq data was downloaded from Phytozome (https://phytozome-next.jgi.doe.gov/info/Ptrichocarpa_v4_1). Data is presented as mean (n=2). The cuttings of *Populus trichocarpa* (Nisqually-1) were potted in 4" X 4" X 5" containers which contain 1:1 mix of perlite and peat. Plants were grown under 16-h/8-h light/dark conditions and were maintained at 20-23°C.
