# Supplementary material for: Agave REVEILLE1 regulates the onset and release of seasonal dormancy in Populus
Source: Plant Physiol. 2022 Dec 22;191(3):1492–504. doi: 10.1093/plphys/kiac588 (PMC10022617; doi:10.1093/plphys/kiac588)
Supplement: kiac588_Supplementary_Data [file kiac588_supplementary_data.zip › Supplemental Table S3_Primers used in this study.pdf]

Supplemental Table S3. Primers used in this study.

Primer name	Primer sequence (5′-3′)
AaRVE1_qPCR_F2	GTATTGAGATACCTCCCCCTCGACC
AaRVE1_qPCR_R2	CTGCCGATAGGACCGAAGTCGGTG
PtrActin-F	TGTTGCCCTTGACTATGAGCAGGA
PtrActin-R	ACGGAATCTCTCAGCTCCAATGGT
